# Mechanistic Analysis of Age-Related Clinical Manifestations in Down Syndrome

**DOI:** 10.3389/fnagi.2021.700280

**Published:** 2021-07-01

**Authors:** Xu-Qiao Chen, Zhuo Xing, Quang-Di Chen, Richard J. Salvi, Xuming Zhang, Benjamin Tycko, William C. Mobley, Y. Eugene Yu

**Affiliations:** ^1^Department of Neurosciences, University of California San Diego, La Jolla, CA, United States; ^2^The Children’s Guild Foundation Down Syndrome Research Program, Genetics and Genomics Program and Department of Cancer Genetics and Genomics, Roswell Park Comprehensive Cancer Center, Buffalo, NY, United States; ^3^Department of Communicative Disorders and Sciences and Center for Hearing and Deafness, University at Buffalo, Buffalo, NY, United States; ^4^Department of Microbiology and Immunology, University of Arkansas for Medical Sciences, Little Rock, AR, United States; ^5^Hackensack-Meridian Health Center for Discovery and Innovation, Nutley, NJ, United States; ^6^Georgetown Lombardi Comprehensive Cancer Center, Washington, DC, United States; ^7^Genetics, Genomics and Bioinformatics Program, State University of New York at Buffalo, Buffalo, NY, United States

**Keywords:** Down syndrome, Alzheimer’s disease, hearing loss, infection, COVID-19, mechanisms

## Abstract

Down syndrome (DS) is the most common genetic cause of Alzheimer’s disease (AD) due to trisomy for all or part of human chromosome 21 (Hsa21). It is also associated with other phenotypes including distinctive facial features, cardiac defects, growth delay, intellectual disability, immune system abnormalities, and hearing loss. All adults with DS demonstrate AD-like brain pathology, including amyloid plaques and neurofibrillary tangles, by age 40 and dementia typically by age 60. There is compelling evidence that increased *APP* gene dose is necessary for AD in DS, and the mechanism for this effect has begun to emerge, implicating the C-terminal APP fragment of 99 amino acid (β-CTF). The products of other triplicated genes on Hsa21 might act to modify the impact of *APP* triplication by altering the overall rate of biological aging. Another important age-related DS phenotype is hearing loss, and while its mechanism is unknown, we describe its characteristics here. Moreover, immune system abnormalities in DS, involving interferon pathway genes and aging, predispose to diverse infections and might modify the severity of COVID-19. All these considerations suggest human trisomy 21 impacts several diseases in an age-dependent manner. Thus, understanding the possible aging-related mechanisms associated with these clinical manifestations of DS will facilitate therapeutic interventions in mid-to-late adulthood, while at the same time shedding light on basic mechanisms of aging.

## Introduction

**Figure 1:**
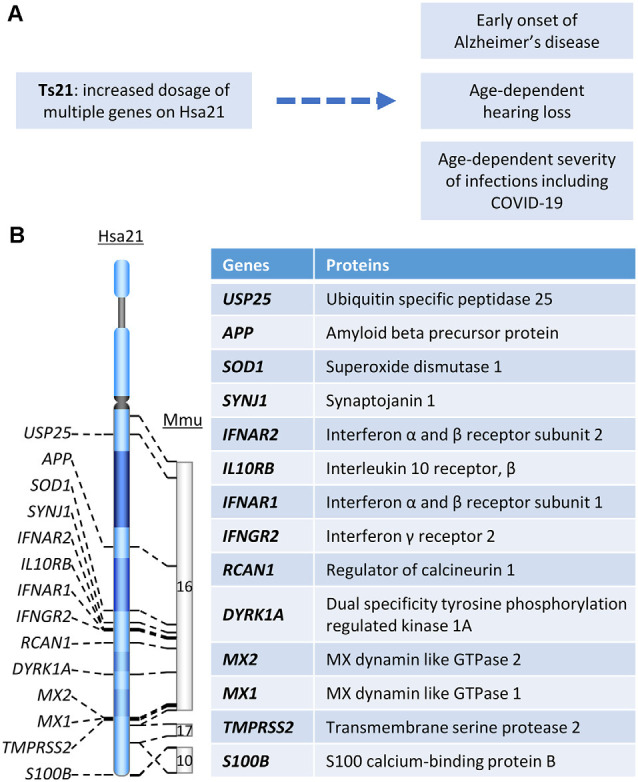
**(A)** The relationship between Hsa21 and age-related clinical phenotypes. **(B)** Hsa21 gene orthologs which play important roles in Down syndrome (DS)-associated dementia and other age-related clinical phenotypes.

Down syndrome (DS), associated with trisomy 21 (Ts21), occurs in 1 in ~800 live births, leading to an estimated 200,000–250,000 people with this condition in the US (Bull, 2020). It is the most common genetic cause of developmental intellectual disability and early-onset Alzheimer’s disease (AD) with very high penetrance (Dierssen, 2012; Strydom et al., 2018; Antonarakis et al., 2020). Children and adults with DS also have an increased incidence of several other important medical conditions that are discussed here. With improved medical care and social support, life expectancy in DS has substantially risen to 60 years, with many living into their 70s (Bittles and Glasson, 2004; Henderson et al., 2007). However, with this increased lifespan, more individuals with DS become affected by age-related clinical phenotypes, a problem that is thought to be aggravated by accelerated biological aging in this syndrome. Thus, research on age-related phenotypes in the DS is becoming increasingly active and important. In this review, we discuss DS-associated AD, age-related hearing loss, bacterial and viral infections including COVID-19, and other age-related medical conditions in DS (Figure 1A) that we are investigating in our laboratories.

## Mechanisms of Alzheimer’s Disease in Down Syndrome

### Alzheimer’s Disease-Related Dementia and Neuropathology in DS

Dementia is defined as a decline in cognitive function sufficient to interfere with a person’s ability to conduct a normal daily life. AD is the most common type of dementia with clinical manifestations including memory loss, language problems, cognitive decline, and behavior dysfunction (Scheltens et al., 2016). AD dementia follows a progressive course in which early subtle changes in memory are followed in time by worsening function, leading to the inability to carry out many facets of daily life, with the disintegration of personality (DeTure and Dickson, 2019). DS, the most common genetic cause of AD, is due to trisomy for all or part of chromosome 21 (Hsa21; Figure 1A). Due to the increased dosage of genes on Hsa21, DS presents with findings related to dysfunction of multiple body systems. Clinical manifestations apparent even in the newborn period are changes in craniofacial anatomy. Compromised cognition and the delays in development of intellectual and behavioral milestones are essentially universal in children (Antonarakis et al., 2020). By age 40, almost all individuals with DS show AD-like neuropathology and by age 56 fully one-half are diagnosed with dementia (Chen and Mobley, 2019a). The prevalence of dementia in DS was reported to range from 30 to 75%; some studies estimate greater than 80% of dementia beyond age 65 (Zigman et al., 1997; Hithersay et al., 2017). Clinical and neuropathological similarities justify the designation of AD in DS (AD-DS).

The neuropathological changes in AD-DS are much like those in non-DS AD, including amyloid plaques and neurofibrillary tangles (NFTs). Amyloid plaques are extracellular accumulations of amyloid derived from Aβ peptides of various lengths, which are products of Amyloid Precursor Protein (APP) processing (Figure 1B, see below; Chen and Mobley, 2019a; Lott and Head, 2019). In AD, however, deposition of Aβ in amyloid plaques routinely fails to show a correlation with dementia, while there is a consistent correlation between disease progression and NFTs. The latter are composed of aberrantly folded and abnormally phosphorylated tau (p-tau; Chen and Mobley, 2019a). A recent study confirms the same pattern for AD-DS. Comparing DS with and without dementia with respect to cortical and striatal plaques and tangles showed that plaques did not predict AD in DS subjects, while abnormal tau aggregation in tangles was correlated with dementia (Perez et al., 2019). The neuropathological features shared between non-DS and DS-associated AD have been recently reviewed (Chen and Mobley, 2019a; Lott and Head, 2019).

### Role of *APP* Triplication in AD-DS

Rare cases of early-onset AD are due to duplication of a small *APP* gene-containing chromosomal segment (Figure 1B), which is evidence that increased *APP* gene dosage is sufficient to cause AD (Cabrejo et al., 2006; Sleegers et al., 2006). The evidence is likewise compelling that increased *APP* copy number is necessary for AD-DS (Prasher et al., 1998; Doran et al., 2017). Neuropsychological and pathological studies in two partial trisomy DS subjects demonstrated sharing of several typical phenotypic features of DS (short stature, Brushfield spots, hearing problems, etc.). Though harboring duplicated segments of varying length, in both cases the *APP* gene was present in two, not three, copies. Both died at advanced age free of dementia and the neuropathological hallmarks of AD (Prasher et al., 1998, Doran et al., 2017). These data converge with those in mouse models to demonstrate the necessity of increased *App* gene dose for AD-relevant phenotypes in DS (Salehi et al., 2006, 2009). Although triplication of other genes in Hsa21 has been explored for an effect on AD-linked neuropathologies in DS mouse models, direct evidence linking any other triplicated gene to AD pathology is as yet lacking. Nevertheless, it is likely that other genes will contribute. For instance, DYRK1A (Figure 1B) was shown to impact APP processing (Branca et al., 2017) and modify tau phosphorylation (Ryoo et al., 2007). Rcan1 can also modulate tau phosphorylation by both decreasing *p*-tau dephosphorylation and increasing tau phosphorylation (Lloret et al., 2011; Figure 1B). Reduction of synaptojanin 1 improved amyloid-induced neuropathology and behavior deficits through accelerating Aβ clearance in one human Swedish APP and FAD (familial AD)-linked PS1 (presenilin-1) double mutant transgenic mouse (Zhu et al., 2013; Figure 1B). Moreover, synaptojanin 1 was also linked to enlargement of early endosome in DS (Cossec et al., 2012).

APP is a type 1 transmembrane protein and can be processed by two pathways: the non-amyloidogenic pathway and the amyloidogenic pathway. In the former, APP is sequentially cleaved by α-secretase to produce the soluble fragment sAPPα and α-CTF (C-terminal APP fragment of 83 amino acids); in the latter, APP is cleaved by β-secretase to form sAPPβ and β-CTF (C-terminal fragment of 99 amino acids). α-CTF is then cleaved by γ-secretase to yield the APP intracellular domain (AICD) and the P3 peptide; cleavage of β-CTF yields the same AICD and Aβ peptides of varying length (Zhang et al., 2011; Chen and Mobley, 2019a). Triplication of *APP* gene in DS predicts increased levels in APP and its products (Nistor et al., 2007; Iulita et al., 2014; Chen et al., 2021). Consistently, reducing *APP* gene dose to two by CRISPR/Cas9 in DS induced pluripotent stem cells (iPSCs)-derived cortical neurons almost normalized the levels of APP, Aβ42, and the Aβ42/40 ratio (Ovchinnikov et al., 2018). APP and Aβ peptide have been linked to tau pathology in several studies (Hardy and Selkoe, 2002; Kwak et al., 2020), however, this conclusion was contested when normalizing *APP* gene dose in DS iPSC-derived neurons did not impact tau hyperphosphorylation (Ovchinnikov et al., 2018). In contrast, we found that treating with Posiphen to normalize APP levels in the Ts65Dn mouse reduced the levels of not only Aβ42 but also p-tau to levels in the brains of 2N (i.e., euploid) mice (Chen X. Q. et al., 2021; Chen, 2021). Considering that protein products of other triplicated genes on Hsa21, including Dyrk1a and Rcan1, have been shown to contribute to tau hyperphosphorylation in DS (Antonarakis et al., 2020), whether or not and to what extent APP and/or its products are linked to tau pathology need further elucidation. Nevertheless, evidence supporting a role for *APP* gene dose in both Aβ and tau-related pathologies were those for the two partial Ts21. Neither of them showed the senile plaques or NFTs typical of the AD-DS brain (Prasher et al., 1998; Doran et al., 2017).

### AD-Associated Features in DS: Endosomal Abnormalities

DS mouse models support research on mechanisms leading to AD-DS (Davisson et al., 1990; Yu et al., 2010a; Herault et al., 2017). Due to *App* gene dosage, APP along with its processing products including CTFs and Aβ peptides is significantly increased in Ts65Dn mice. Normalizing *App* gene copy number in these mice (Ts65Dn^APP++-^) restored the levels of APP and its CTFs (Salehi et al., 2006). It was noted that in Ts65Dn^APP++-^ mouse, reduced NGF transport in basal forebrain cholinergic neurons (BFCNs), as well as the BFCN atrophy, were both significantly improved, pointing to defective retrograde signaling of NGF as contributing to BFCN loss (Salehi et al., 2006). Further studies linked the deficits in NGF axonal transport to abnormal early endosome pathologies including endosome enlargement and Rab5 hyperactivation (Xu et al., 2016).

**Figure 2:**
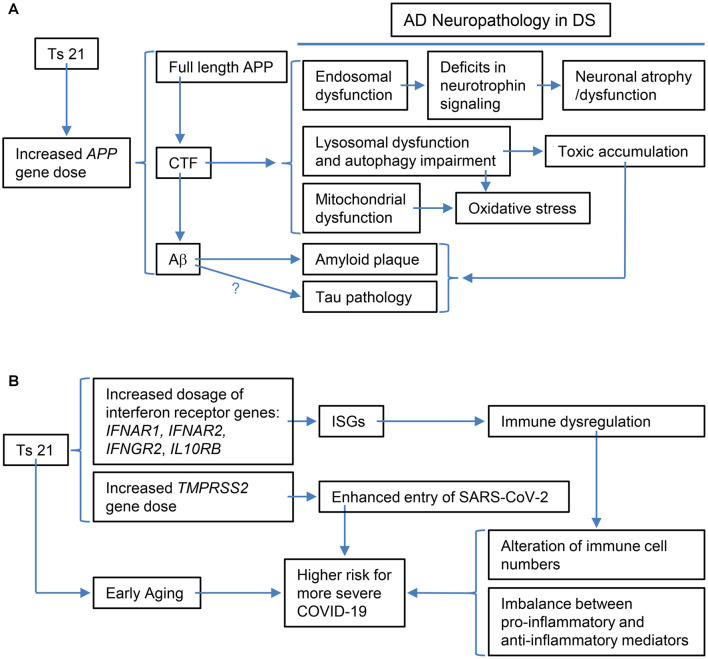
Schematic representations of the specific examples of the relationship between the triplication of Hsa21 or Hsa21 gene ortholog(s) and its phenotypic consequences at various levels. **(A)** The triplication of the *APP* ortholog and its impacts on Alzheimer’s disease (AD)-related phenotypic features: increased *APP* gene dose in DS leads to increases in full-length APP protein and its products, including CTF (α-CTF and β-CTF) and Aβ peptides of varying length. Accumulating evidence points to β-CTF as driving endosomal dysfunction, lysosomal dysregulation, autophagy impairment as well as mitochondrial dysfunction. Abnormal early endosomes may contribute to deficits in the retrograde axonal transport of neurotrophic signaling in several neuron populations, including BFCNs, thus compromising their trophic support and leading to neuronal dysfunction and atrophy. Lysosomal dysregulation and autophagy impairment can allow for the build-up of toxic proteins and induce oxidative stress due to failed clearance of organelles, including mitochondria. β-CTF accumulation could also lead to mitochondrial dysfunction. In addition, increased Aβ peptides contribute to amyloid plaque formation; evidence supports a role for Aβ in tau pathology, but as yet there is no direct demonstration for this in DS. Autophagy-lysosomal system dysfunction also contributes to amyloid and tau pathologies. **(B)** The triplication of Hsa21 or Hsa21 gene orthologs and its impacts on the immune system and COVID-19 in the DS population: The dosage increase of the four interferon receptor genes in Ts21, *IFNAR1*, *IFNAR2*, *IFNGR2*, and *IL10RB*, up-regulates expression of interferon-stimulated genes (ISGs), which in turn results in immune dysregulation, including alteration of immune cell numbers as well as an imbalance between pro-inflammatory and anti-inflammatory mediators. These changes in DS are likely related to a higher risk for more severe COVID-19, which may also be contributed by the dosage increase of *TMPRSS2* and early aging. BFCNs, basal forebrain cholinergicneurons; α-CTF, C-terminal APP fragment of 83 amino acids.

Apparent enlargement of early endosomes due to excessive activation of small GTPase Rab5 is another shared hallmark of AD in non-DS and DS, one that emerges decades before the appearance of amyloid plaques and NFTs (Cataldo et al., 2000; Chen and Mobley, 2019b). A recent study using ultrastructural methods found that endosomes were clustered in fibroblasts and DS induced pluripotent stem cells (iPSCs)-derived cortical neurons from DS individuals and BFCNs of the Ts65Dn DS mouse model (Botte et al., 2020). They interpreted these findings as evidence that clustering of endosomes is responsible for their apparent enlargement. Whether enlarged or clustered, the significant upregulation in the levels of active Rab5 (GTP-loaded Rab5), which drives endosome fusion, support the importance of changes in early endosomes in DS and AD-DS, as well as in non-DS AD (Xu et al., 2016; Chen X. Q. et al., 2021). Evidence from multiple studies using APP knockdown, the Ts65Dn^APP++-^ mouse, and APP/β-CTF overexpression support that increased *APP* gene dose induces early endosome enlargement and point to β-CTF as the major driver of this change (Salehi et al., 2006; Jiang et al., 2010; Kim et al., 2016; Xu et al., 2016; Figure 2A).

Importantly, β-CTF mediated atrophy was prevented by a dominant negative version of Rab5 pointing to an essential role for Rab5 hyperactivation in this process (Xu et al., 2016). The roles of *App* gene dose in abnormal endosome phenotypes and deficient axonal transport of neurotrophin signaling were further supported by a recent study in which Posiphen reversed Rab5 hyperactivation, restored the size of early endosomes, and restored retrograde axonal transport of neurotrophins in primary cortical Ts65Dn neurons and of neurotrophin signaling in the Ts65Dn brains, with the drug acting, at least in part, through reducing the levels of APP and CTFs in a translation-dependent manner (Chen X. Q. et al., 2021). These data are evidence that increased *APP* gene dose in DS, along with increased levels of APP and its products, including β-CTF, acts to induce Rab5 hyperactivation and reduce retrograde transport of neurotrophin signaling, thus linking *APP* gene dose to neurodegeneration.

Early endosomes are upstream of late endosomes and multivesicular bodies (MVB) whose contents are then moved to lysosomes (Grant and Donaldson, 2009). The MVB is a specialized endosome characterized by intraluminal vesicles (ILVs) that bud inward into the endosomal lumen. If an MVB fuses with the plasma membrane, the ILVs can be released into the extracellular space as exosomes (Hanson and Cashikar, 2012). Suggested to serve as a buffering mechanism to alleviate endosomal dysfunction, exosome release from MVBs is increased in DS brains and Ts21 fibroblasts as well as in DS mouse models, possibly through the enhanced expression of CD63 which regulates exosome biogenesis (Gauthier et al., 2017). Upregulation of intraluminal vesicles (ILVs) in MVBs was reported in the Ts2Cje model of DS (D’Acunzo et al., 2019). It is noteworthy that exosomes contain APP-derived metabolites raising the possibility of a protective role in removing toxic products, including the C-terminal fragment of 99 amino acids (β-CTFs) and Aβ species (Perez-Gonzalez et al., 2020). If exosomes play this role, they may enhance Aβ clearance through a microglial cell-dependent pathway. However, as a mediator of intercellular communication exosomes might also propagate and spread Aβ- and tau-related pathologies within neuronal circuits (Mathews and Levy, 2019). Dysregulation of the retromer complex system, which sorts and traffics proteins from endosomes to the trans-Golgi network or the cell surface, was also recently reported to be an early event in the development of AD pathology and cognitive decline in DS (Curtis et al., 2020). Finally, there is evidence pointing to lysosome-autophagy deficits in DS (reviewed in Nixon, 2017; Colacurcio et al., 2018).

In addition to disrupting early endosomes, β-CTF was also shown to mediate APP-induced dysfunction of the lysosomal system through affecting the expression and maturation and/or activity of lysosomal enzymes including cathepsin D, possibly through APP-induced abnormal lysosomal acidification in DS fibroblasts and the Ts2 mouse model (Jiang et al., 2019). These findings were supported in studies in an AD mouse model (3xTgAD) and adeno-associated viral-mediated β-CTF-infected mice (Lauritzen et al., 2016; Figure 2A). In the latter, the aggregation of β-CTF in endosomal-autophagic-lysosomal vesicles caused disrupted lysosomal proteolysis and autophagic impairment (Lauritzen et al., 2016).

### AD-Associated Features in DS: Mitochondrial Deficits

Mitochondria are membrane-bound organelles that supply ATP as a source of energy for cell function, and mitochondrial dysfunction is another hallmark of DS (Izzo et al., 2018). Several lines of evidence suggest that impaired activity of peroxisome proliferator-activated receptor gamma coactivator 1α (PGC-1α) and hyperactivation of the mammalian target of rapamycin (mTOR) kinase contribute to this dysfunction (Mollo et al., 2020). Mitophagy, which clears damaged mitochondria, was recently demonstrated to be deficient in DS fibroblasts leading to the accumulation of damaged mitochondria. This study showed that reductions in PARKIN and PINK1 impaired initiation of mitophagy, together with hyperactivation of mTOR (Bordi et al., 2019). Consistently, inhibition of mTOR with rapamycin reduced APP levels and attenuated the neurodegenerative phenotypes linked to APP overexpression in Ts65Dn mice (Tramutola et al., 2018). A role of “mitovesicle”, a recently defined extracellular vesicle containing mitochondrial components, was suggested as further reflecting mitochondrial pathology in DS (D’Acunzo et al., 2021).

Accumulation of damaged mitochondria is associated with oxidative stress (Bordi et al., 2019), and recent studies linked mitochondrial defects and oxidative stress with insulin resistance in the development of AD pathology in DS (Lanzillotta et al., 2021). Oxidative stress can drive protein oxidation and the formation of protein aggregates (Lanzillotta and Di Domenico, 2021) which are then cleared by protein quality control systems. Among the latter, the integrated stress response (ISR) downregulates protein synthesis to respond to stress. ISR was shown to be activated in the brains of Ts65Dn DS mouse models and DS patients, and suppression of the ISR reversed changes in translation and rescued deficits in synaptic plasticity and long-term memory (Zhu et al., 2019).

A possible link between β-CTF and abnormal mitochondrial structure and function as well as mitophagy defects was supported. The β-CTF was demonstrated to induce mitochondrial morphology alterations and overproduction of mitochondrial reactive oxygen species and to elicit mitophagy failure (Vaillant-Beuchot et al., 2021; Figure 2A). Moreover, β-CTF was shown to accumulate in mitochondria-associated membranes and to regulate extracellular cholesterol uptake and trafficking (Montesinos et al., 2020). Though not documented in the context of DS, accumulation of β-CTF has also been linked to inflammation, synaptic dysfunction as well as behavior deficits in the mouse. The unique contribution of β-CTF to AD pathology has been reviewed (Checler et al., 2021). In addition, the contribution of *APP* gene dose-induced increases in Aβ peptides including those toxic Aβ oligomers has been extensively explored and reviewed (Head et al., 2016; Chen and Mobley, 2019a).

### AD-Associated Features in DS: Neuroinflammation

In addition, neuroinflammation, involving microglial cells and astrocytes, is presumed to represent a response to Aβ and tau pathologies, with the inflammatory events and the transcriptional pathways thus engaged being viewed as contributing to brain pathology in AD (Wilcock, 2012; Kinney et al., 2018; Nott et al., 2019). A recent neuropathology study characterized an early and evolving neuroinflammatory phenotype across the lifespan in DS, with a higher microglial soma size-to-process length ratio and increased levels of several inflammatory cytokines observed in autopsy brains of children and young adults with DS (Flores-Aguilar et al., 2020). Consistently, in the Dp(16)1Yey mouse model of DS microglia were hyperactivated, with increased pro-inflammatory cytokine levels and altered interferon signaling in the hippocampus, and with decreased spine density and activity of hippocampal neurons and hippocampus-dependent cognitive behavioral deficits (Pinto et al., 2020). Both pharmacological depletion of defective microglia and anti-inflammatory treatment with acetaminophen rescued the deficits in these mice, suggesting a link between aberrant microglia and cognitive dysfunction in Dp(16)1Yey (Pinto et al., 2020). But how such treatments can overcome the impact of important triplicated genes, such as *Dyrk1a*, in the mouse model remains to be revealed, in light of the relationship between *Dyrk1a* and inflammation (Latour et al., 2019).

Unlike non-DS AD, the brain in DS harbors triplication of many inflammation-related genes, including *SOD1*, *S100B*, and genes encoding multiple interferon receptors and several interferon target genes on Hsa21, raising the possibility that DS constitutes a unique environment for the inflammatory signals characteristic of AD neuropathology (Figure 1B; Wilcock, 2012). Interestingly, USP25, a deubiquitinating enzyme encoded on Hsa21 (Figure 1B), has been linked to aberrant microglia activation as well as deficient synaptic and cognitive function. Both genetic ablation and pharmaceutical inhibition of USP25 reduced microglia-mediated neuroinflammation and restored synaptic and cognitive function in the 5xFAD mouse model of AD (Zheng et al., 2021).

## Hearing Loss in Down Syndrome

DS is characterized by a variety of craniofacial anomalies such as softening of the tissue above the larynx (laryngomalacia) and narrowing of the trachea, conditions that contribute to obstructive sleep apnea, voice disorders, and articulatory impairments (Vicente et al., 2020). Individuals with DS tend to have small ears suggestive of the potential for hearing impairments (Aase et al., 1973) The incidence of hearing impairment is relatively high in DS (Keiser et al., 1981; Roizen et al., 1993; Laws and Hall, 2014), with hearing loss ranging from 34% to 78% for children (Shott et al., 2001; Yam et al., 2008; Raut et al., 2011). The nature of this hearing loss and its developmental progression is varied and may depend on the nature of the craniofacial anomalies (Diefendorf et al., 1995).

## Conductive Hearing Loss

DS generally impairs the transmission of sound through the external ear and middle ear giving rise to what is referred to as a conductive hearing loss—which is characterized by a loss in hearing sensitivity over a broad range of frequencies, unlike sensorineural hearing loss that preferentially affects the high frequencies. In some cases, the craniofacial disorder involves a malformation of the external ear or substantial narrowing of the ear canal. In severe cases, the ear canal can be occluded (atresia) which attenuates sound transmission to the cochlea where the sensory hair cells are located (Diefendorf et al., 1995). The maximum conductive hearing loss is approximately 40 dB.

The medial end of the ear canal terminates at the tympanic membrane, a thin, translucent membrane that separates the external ear from the middle ear. The middle ear space, which consists of an air-filled cavity in the mastoid bone, is connected to the pharyngeal cavity by the Eustachian tube. The Eustachian tube serves to equalize pressure in the middle ear space with the external pressure in front of the tympanic membrane. The middle ear ossicles composed of three extremely small bones (malleus, incus, and stapes) slightly amplify the sound-induced vibrations relayed from the tympanic membrane to the cochlea. One of the most common problems in DS is Eustachian tube malformation that can result in recurrent middle ear infections that can lead to otitis media with effusion. The fluid in the middle ear greatly attenuates the transmission of sound to the cochlea.

Otoscopy can be used to visualize inflammation of the tympanic membrane, fluid buildup in the middle ear, and rupture of the membrane. Middle ear function can be evaluated with impedance-admittance audiometry which involves inserting a specialized probe into the external ear. The device can detect abnormal middle ear pressure, tympanic membrane rupture, immobility of the tympanic membrane caused by fluid in the middle ear, or hypermobility of the tympanic membrane caused by disarticulation of the middle ear ossicles. Among young DS patients with hearing loss, more than 80% were attributed to conductive hearing loss, mostly due to middle ear effusion (Schwartz and Schwartz, 1978; Austeng et al., 2013). Other factors implicated in conductive loss included immobility or deformities to the middle ear ossicles (Balkany et al., 1979). Histological analysis of temporal bones from DS patients revealed numerous differences in the dimensions of the middle ear space and shortening of the length of the cochlea (Igarashi et al., 1977; Harada and Sando, 1981).

## Aging and Sensorineural Hearing Loss

Hearing loss in DS can also result from damage to the sensory and neural structures in the cochlea. Some have reported that < 5% of DS patients suffer from sensorineural hearing loss (De Schrijver et al., 2019) whereas others estimate 53% of the cases are of sensorineural origin (Glovsky, 1966; Brooks et al., 1972). One factor that could contribute to the diversity of results is the age of the subjects. Adults are much less likely than children to develop a middle ear infection but are more likely to have sensorineural hearing loss due to aging (presbyacusis) or other factors (Buchanan, 1990).

Most age-related disorders in DS begin around 40, approximately 20 years earlier than the general population (Martin, 1978; Steingass et al., 2011; Carfi et al., 2014; Glasson et al., 2014). To assess the rate of age-related hearing loss in DS, subjects with conductive or mixed hearing loss were excluded from the analysis (Buchanan, 1990). Sloping, high-frequency sensorineural hearing loss characteristic of age-related hearing loss was evident in young DS subjects. Hearing losses in DS reached 90 dB HL in the 51–60 years age group, compared to 50 dB HL in age-matched controls. Age-related hearing loss occurred 20–30 years earlier in DS than controls, indicating that DS accelerates age-related hearing loss, consistent with other reports (Picciotti et al., 2017).

Histological analysis of temporal bones of elderly DS subjects revealed excessive bone growth and blockade of the canals in the bone through which the peripheral auditory nerve fibers of the spiral ganglion travel out to contact the sensory hair cells in the cochlea (Krmpotic-Nemanic, 1970). Others have used computed tomography to evaluate adult DS subjects, and while no ossicular malformations were detected, vestibular and other inner ear malformations were observed in nearly half of DS subjects (Saliba et al., 2014).

## Central Auditory Dysfunction

Hearing problems in DS may also relate to auditory structures in the central nervous system. Brain imaging studies indicate total brain volume is reduced by roughly 20% in DS subjects, with major reductions in the brainstem, hippocampus, temporal lobe, and cerebellum (Fujii et al., 2017; Rodrigues et al., 2019). These changes may be due stunted dendrite growth and atrophy (Becker et al., 1991), changes that could disrupt sound-evoked neural activity in the central auditory pathway. The auditory brainstem response (ABR) is an electrophysiological technique used to assess sound-evoked neural activity in the brainstem. In humans, the ABR waveform consist of five positive and negative peaks, numbered I, II, III, IV and V, occurring 1–7 ms following stimulus onset. Among young adults, the amplitudes of the click-evoked peaks in the ABR waveform were smaller in DS than controls. Stimulus intensities needed to elicit the ABR responses were higher in DS than in controls, indicative of hearing loss and the slope of the latency vs. intensity function in DS was steeper than in controls consistent with a high-frequency sensorineural hearing loss (Widen et al., 1987). In DS infants 12 months or younger, the absolute latencies of the ABR peaks were shorter than normal and the slope of the latency-intensity functions was steeper than normal (Folsom et al., 1983). Others have reported that the interval between peaks I–II and III–IV were shorter than normal in DS, whereas the IV–V interval was longer than normal (Squires et al., 1980) possible due to a smaller brain size or other brainstem abnormalities.

Animal models can be used to explore the mechanisms underlying conductive, sensorineural, and age-related hearing loss in DS. Most of our efforts and those of others have mainly focused on identifying genomic regions associated with the development of otitis media in DS based on analysis of mouse models triplicated for different Hsa21 orthologous regions (Han et al., 2009; Bhutta et al., 2013; Chen et al., 2013). In Ts65Dn mice, which share many phenotypic characteristics with DS, middle ear effusions were present in nearly 75% of these mice whereas effusions were rare in WT controls. The middle ears in these mice showed varying degrees of inflammation, thickening of the middle ear mucosae, and the presence of goblet cells and pathogenic bacteria (Han et al., 2009).

Additional efforts are underway to model and mechanistically understand the origins of hearing disorders in various DS mouse models. One of the problems associated with many mouse models is that they were developed on background strains that exhibit early-onset age-related hearing loss such as the widely used C57BL strain. This strain carries a single nucleotide variant (SNV) of the cadherin 23 gene (Cdh23^c.753A^) that causes age-related hearing loss in wild-type C57BL mice (Johnson et al., 2017). Therefore, this variant should be removed to investigate age-related hearing loss in DS.

The ABR, widely used to investigate age-related hearing loss in mice (Boettcher, 2002; Konrad-Martin et al., 2012; Johnson et al., 2017; Grose et al., 2019), can also be used to assess auditory function in DS mouse models (Widen et al., 1987; Johnson et al., 2017). Tone burst-evoked ABR provides a useful method for assessing function at different frequencies and estimating the amount of hearing loss at different frequencies. In preliminary studies, we have measured ABR thresholds in a small cohort of DS mice (Yu et al., 2010b) backcrossed for four generations on CBA/J mice a strain that shows little evidence of age-related hearing impairment until extremely late in life so B6-specific age-related Cdh23^c.753A^ allele has been converted to CBA/J-specific Cdh23^c.753G^ (Spongr et al., 1997; Zheng et al., 1999; Han et al., 2016). Our preliminary results from 3-month-old mice revealed slightly elevated (~25 dB) ABR thresholds and slightly reduced ABR amplitudes in DS mice compared to WT mice. Thresholds in DS mice were elevated over a wide range of frequencies, results indicative of a conductive hearing loss (Bhutta et al., 2013). However, histological results are needed to assess the status of the middle ear and cochlea. To determine if age-related hearing loss is more rapid in DS mice, ABR thresholds in DS and WT will be monitored to determine the rate at which age-related hearing loss develops. Afterward, the cochlea will be evaluated to determine the percentage of missing outer hair cells and inner hair cells. Outer hair cell and inner hair cell losses in DS and WT mice will be compared to determine if age-related sensory cell losses are more severe and develop more rapidly in DS mice compared to WT mice (McFadden et al., 1999; Johnson et al., 2010).

## Is Hearing Loss Related to Cognitive Decline and Dementia in DS?

Although hearing loss is mainly considered a sensory disorder, the communication difficulties that it imposes often contribute to social isolation and depression. Recent meta-analyses of human data indicate that age-related hearing loss has a significant association with adverse health outcomes. One of the most unexpected findings was that hearing loss was positively correlated with age-related cognitive decline, cognitive impairment, and dementia and there was a non-significant trend for an association with AD. Vascular disorders, social isolation, or impaired verbal communication were suggested as contributing factors (Lin et al., 2011; Su et al., 2017).

Animal models could provide mechanistic insights on the contribution of hearing loss to cognitive decline, dementia, and AD. Recent animal studies have explored the relationship between noise-induced hearing loss and cognitive impairment using hippocampal-dependent maze learning tasks that assess the acquisition and retention of spatial memory. Hearing loss was associated with a significant decline in the acquisition of spatial memory and deficits in spatial memory retention (memory consolidation; Liu et al., 2016; Park et al., 2016; Manohar et al., 2020). Importantly, these deficits were associated a significant decline in hippocampal neurogenesis (Kraus et al., 2010; Newman et al., 2015; Manohar et al., 2020) and an increase in p-tau protein and lipofuscin in the hippocampus (Park et al., 2018). These results are consistent with the view that hearing loss contributes to cognitive decline, but further studies are needed to assess if hearing loss contributes to the development of dementia and AD.

## Aging, Immunity, and Infections in Down Syndrome

### DS-Associated Immune Dysregulation: Role of Interferon Pathways

As reviewed by us recently, people with DS are at increased risk of various viral and bacterial infections, while at the same time having a markedly increased susceptibility to autoimmune disorders (Yu et al., 2020). Here we further discuss potential mechanisms of dysregulated immunity in DS, including a role for premature aging, and complete the discussion with some thoughts on relevance to the current COVID-19 pandemic (Figure 2B).

The immune system defends against invading pathogens with four main steps: recognize, alert, destroy, and clear (Mueller et al., 2020). The normal immune response to infection comprises two parts: the innate and the adaptive immune systems (Bajaj et al., 2020). The innate immune system is the first line of defense against invading microorganisms and ultimately regulates the adaptive immune response generated by both T and B cells. Viral infections are first detected in host cells by specific pathogen recognition receptor (PRR) sensor molecules, such as the toll-like receptors (TLRs), the retinoic acid-inducible gene 1 protein (RIG-I), and melanoma-differentiation-associated protein-5 (MDA5), which initiate cascades of signaling events that lead to the expression of immune-regulatory and antiviral genes, such as interferons (IFNs) and their downstream target genes (Yoneyama and Fujita, 2010; Murira and Lamarre, 2016).

Indeed, one of the keys linking between Ts21 and immune dysfunction is IFNs and their receptors. Among six IFN receptors, four are encoded by genes clustered on chromosome 21: *IFNAR1* and *IFNAR2*, coding for type I IFN receptors; *IFNGR2*, coding for type II IFN receptor, and *IL10RB*, coding for the receptor required for type III IFN ligands and cytokines like interleukin-10 (IL-10), IL-22, and IL-26 (Figures 1B, 2B; De Weerd and Nguyen, 2012; Espinosa, 2020). Over-expression of these IFN receptors in individuals with DS leads to hyperactivation of IFN signaling in multiple immune and non-immune cell types and a significantly higher level of key cytokines, including C-reactive protein (CRP), IL-2, IL-6, IL-10, tumor necrosis factor-α (TNF-α), interferon γ-induced protein 10 (IP-10), and monocyte chemoattractant protein-1 (MCP-1; Sullivan et al., 2016, 2017; Araya et al., 2019; Espinosa, 2020). IFNs are essential for antiviral immunity and play a key role in immediate antiviral responses to viral infection in an autocrine and paracrine manner through IFN receptors (IFNARs) signaling and subsequent induction of hundreds of interferon-stimulated genes (ISGs; e.g., *MX1*) to inhibit viral replication and spread (Bajaj et al., 2020; Nikolich-Zugich et al., 2020). IFNs also play an important role in immune modulation and inflammation, as type I IFNs (i.e., IFN-a/b) are often considered double-edged swords as both proinflammatory and anti-inflammatory cytokines. Type I IFNs have been associated with promoting several inflammatory and autoimmune diseases (Hall and Rosen, 2010; Barrett et al., 2020) and have also been successfully used for treatment of inflammatory and autoimmune diseases, such as multiple sclerosis (Kieseier, 2011). Interestingly, several downstream target genes of IFN signaling, including *MX1* and *MX2*, are present on Hsa21 (Figure 1B) and the *MX1* gene was found to be over-expressed to high levels in Ts21 fibroblasts when these cells entered replicative senescence (Li et al., 2006), presumably reflecting the known relationship of *MX1* expression and type I IFN signaling to DNA damage and telomere erosion in cell senescence (Frisch and Macfawn, 2020), aggravated by the increased gene dosage of both the upstream and downstream components of the IFN pathway in the cells with Ts21.

IFN signaling hyperactivation in DS subjects caused by overexpression of the interferon receptors and target genes is linked with chronic immune dysregulation, which has been demonstrated by an abundance of evidence and can be characterized by being more predisposed to bacterial infection in the respiratory tracts, weaker response to antibody, and high level of autoantibodies (Ram and Chinen, 2011; Espinosa, 2020; Gensous et al., 2020). Such an immune dysregulation also results in an imbalance between proinflammatory and anti-inflammatory mediators (Hadjadj et al., 2020; Figure 2B). At the molecular and cellular levels, dysregulation of immunity in DS is frequently associated with a proinflammatory tendency when compared with the general population, even in the absence of any detectable infection. The tendency is reflected by: (A) changes in the number of different types of immune cells (monocytes, dendritic cells, nature kill cells, neutrophils, and T and B cells); (B) overproduction of proinflammatory cytokines, including TNF-α and IL-6 which, relevant to the discussion below, are key predictors of deteriorating health conditions in COVID-19 (Chen et al., 2020; Hadjadj et al., 2020); (C) functional inhibition of the suppressors, like regulatory T cells (Tregs), a key factor to suppress immune response after clearing of viral pathogens (Cetiner et al., 2010; Araya et al., 2019; Espinosa, 2020; Huls et al., 2021).

### Aging and Alteration of the Immune System in DS

The above observation raises the topic of early or accelerated aging in DS. Adults with DS experience certain components of premature aging earlier than the general population, with some typical features including wrinkled skin, gray hair, hearing loss, declining immune function, and increased autoimmune diseases. Using a methodology pioneered by Steve Horvath (Horvath, 2013), DNA methylation patterns can be utilized as an “epigenetic clock” that correlates with chronological age and may reflect underlying biological aging. Both Horvath and his collaborators (Horvath et al., 2015) and our group (Mendioroz et al., 2015; Yu et al., 2020) examined epigenetic aging in human DS, using separate sample sets including blood cells (total leukocytes, and in our second study purified T lymphocytes) and brain tissues. Both datasets reveal rapid aging of CpG methylation patterns during fetal and early postnatal development in DS, leading to a higher “set point” of epigenetic age established by young adulthood, followed by maintenance of this methylation age differential (older in DS than controls), without further acceleration of the difference, throughout adult life (Mendioroz et al., 2015; Yu et al., 2020). Because of the aforementioned association with immune dysregulation and premature aging, it has been proposed that the DS phenotype may include immunosenescence (Gensous et al., 2020).

### COVID-19 in DS

COVID-19, caused by the SARS-CoV-2 coronavirus, is currently a major and too often lethal disease worldwide. Since the clinical outcomes of COVID-19 largely depend on the severity of inflammation and differ strikingly by age (more severe in the elderly), studying this disease in DS may prove to be highly informative. The impacts of COVID-19 on individuals with DS were illustrated by a recent international survey initiated by the Trisomy 21 Research Society and the UK ISARIC4C. One thousand forty-six cases of COVID-19 patients with DS from April to October, 2020 were analyzed, and the data were compared with the hospitalized COVID-19 patients with or without DS in the UK ISARIC4C survey. Based on this study, when compared with non-DS COVID-19 patients, COVID-19 patients with DS exhibited more severe symptoms and were around three times more likely to die. Remarkably, the mortality rates increased rapidly in patients with DS older than 40 years, which resembles the mortality rate for patients without DS at ages over 60. The data in the report indicated that individuals with DS, especially those older than 40 years, are more vulnerable and at higher risk for hospitalization and death due to COVID-19 (Huls et al., 2021).

Several factors discussed in the above sections may contribute to this phenomenon. On the one hand, IFN hyperactivity in DS may present stronger defenses against the virus during the initial stage after exposure to SARS-CoV-2, which may dampen the virus load in the infected site. On the other hand, over-production of the proinflammatory IFNs can overwhelm the system, leading to the so-called cytokine storm and inflammation. Induction of IFNs by coronavirus infection has been proposed as a potential mechanism by which coronavirus establishes persistent infection in cells in the central nervous system (Li et al., 2010; Liu et al., 2011). It remains to be seen, however, whether SARS-CoV-2 establishes persistent infection more readily in individuals with DS as compared to the general populations. Furthermore, several other factors may contribute to this balancing act: (A) Transmembrane protease serine 2 (TMPRSS2) primes the S protein of the SARS-CoV-2 virus to bind to its receptor, angiotensin-converting enzyme 2 (ACE2), during infection (Hoffmann et al., 2020; De Toma and Dierssen, 2021) and *ACE2* is an IFN-stimulated gene (Ziegler et al., 2020). Since the *TMPRSS2* gene is on Hsa21, its triplication leads to its overexpression in DS, which in turn enhances the entry of the coronavirus into host cells (Figures 1B, 2B; De Toma and Dierssen, 2021); (B) SARS-CoV-2 appears to induce low levels of type I and type III IFN responses and elevate IL-6 expression (Blanco-Melo et al., 2020), which might delay local IFN responses and thus enable SARS-CoV-2 to evade recognition and attack by immune cells in order to sustain its replication (Acharya et al., 2020). Recent data demonstrated that, different from mild-to-moderately ill patients, severely ill patients showed a highly impaired type I IFN response and elevated level of TNF-α and IL-6 (Hadjadj et al., 2020); and (C) Aging impaired and delayed the production of type I IFNs (Bajaj et al., 2020) and DS is associated with premature aging, which could be an explanation for initial findings of a higher risk of mortality of DS patients with COVID-19 at ages over 40 years (Huls et al., 2021). On the other hand, as recently proposed, the unusual IFN hyperactivation-related immune dysregulation in DS might in theory also contribute to more frequent cytokine storms induced by SARS-CoV-2 infection in individuals with DS (Bajaj et al., 2020). Cytokine storm is considered as one of the major causes of acute respiratory distress syndrome (ARDS) and multiple organ failure (Ye et al., 2020). Consequently, COVID-19 patients with DS may develop more severe complications and have a higher mortality rate, as illustrated in the aforementioned study (Huls et al., 2021; Figure 2B).

In addition, DS is associated with other comorbidities, such as obesity, diabetes, hypotonia, obstructive sleep apnea, craniofacial dysmorphogenesis, congenital heart defects, and gastroesophageal reflux (Antonarakis et al., 2020; Startin et al., 2020; Huls et al., 2021), which may lead to a higher probability of developing more severe symptoms and elevated mortality when infected with SARS-CoV-2 (Espinosa, 2020). Premature aging, discussed in the preceding section, is possibly one of the important risk factors for developing severe COVID-19 cases among DS individuals (Horvath et al., 2015; Hithersay et al., 2019; Gensous et al., 2020; Yu et al., 2020; Chen Y. et al., 2021; Huls et al., 2021). Lastly, medical complications from COVID-19 developed in approximately 60% of patients with DS and increased with age, which include viral pneumonia (36%), acute respiratory distress syndrome (34%), and secondary bacterial pneumonia (17%; Huls et al., 2021). Compared with the general population, pulmonary complications were frequently presented with significantly higher mortality in the DS population (Huls et al., 2021). It is reasonable to link these complications with an increased incidence of respiratory tract infections in DS, which is associated with other DS-related abnormalities, such as aberrant airway anatomy and physiology, hypotonia, aspiration, and dysphagia (Bloemers et al., 2010). Moreover, IFN hyperactivity and elevated IL-10 level may play a role in these complications, particularly in secondary bacterial pneumonia in DS patients (Espinosa, 2020).

### Modeling and Analysis of Interplays Between Ts21 and SARS-CoV-2

Understanding of the impacts of various factors on disease processes of COVID-19 patients with DS has just been begun. Additional knowledge will be gained by the generation and analysis of model systems. Because of species-specific differences, mouse ACE2 protein does not serve as a effective receptor for SARS-CoV-2. Transferring a human *ACE2* transgenic allele, such as K18-hACE2, to a mouse model of DS is required. The compound models could be used to ascertain similarities and differences with human COVID-19, and the effects of the DS-mimicking genetic background, which include determination of whether viral replication kinetics are altered in the DS-mimicking genetic background and assessment of age-dependency of COVID-19 disease severity. The models can also be used to understand the impacts of individual genes, such as *TMPRSS2* and *MX1* by normalizing their gene dosages in the compound mutants.

Aging has important effects on various organ systems in mammals, and accelerated aging in DS individuals illustrates such importance, which is reflected in early-onset AD and hearing loss as well as more severe COVID-19 symptoms at younger ages. A better understanding of the mechanisms underlying these phenomena in individuals with DS and in animal models will enhance our abilities to provide more effective interventions to improve the quality of the lives of this special population.

## Author Contributions

YY and WM conceived the project. All authors contributed to the article and approved the submitted version.

## Conflict of Interest

WM is a patent holder of a potential treatment for Alzheimer disease held under his employer, the University of California, San Diego. The remaining authors declare that the research was conducted in the absence of any commercial or financial relationships that could be construed as a potential conflict of interest.

## Abbreviations

ABR: auditory brainstem response
ACE2: angiotensin-converting enzyme 2
AD: Alzheimer’s disease
AD-DS: Alzheimer’s disease in Down syndrome
AICD: APP intracellular domain
APP: Amyloid Precursor Protein
ARDS: acute respiratory distress syndrome
β-CTF: C-terminal APP fragment of 99 amino acids
BFCN: basal forebrain cholinergic neurons
CRP: C-reactive protein
DS: Down syndrome
FAD: familial Alzheimer’s disease
Hsa21: human chromosome 21
IFN: interferon
IFNAR: interferon-α/β receptor
IL-10: interleukin-10
ILVs: intraluminal vesicles
IP-10: interferon γ-induced protein 10
iPSC: induced pluripotent stem cells
ISG: interferon-stimulated gene
ISR: integrated stress response
MCP-1: monocyte chemoattractant protein-1
MDA5: melanoma-differentiation-associated protein-5
mTOR: the mammalian target of rapamycin
MVB: multivesicular bodies
NFTs: neurofibrillary tangles
p-tau: phosphorylated tau
PGC-1α: peroxisome proliferator-activated receptor gamma coactivator 1α
PRR: pathogen recognition receptor
PS1: presenilin-1
RIG-I: retinoic acid-inducible gene 1 protein
SNV: single nucleotide variant
TLRs: toll-like receptors
TMPRSS2: transmembrane protease serine 2
TNF-α: tumor necrosis factor-α
Ts21: trisomy 21
α-CTF: C-terminal APP fragment of 83 amino acids.
